# Novel NR5A1 variants associated with hypospadias and disorders of sex development: A series case report of 4 patients

**DOI:** 10.1097/MD.0000000000046626

**Published:** 2025-12-12

**Authors:** Xiangwen Peng, Jiancheng Zu, Sifeng Wang, Qingqing Hu

**Affiliations:** a Changsha Central Hospital, Changsha, Hunan, China; b Hunan Provincial Key Laboratory of Regional Hereditary Birth Defects Prevention and Control, Changsha Hospital for Maternal & Child Health Care Affiliated to Hunan Normal University, Changsha, Hunan Province, China; c Hunan Children’s Hospital, Hunan, China.

**Keywords:** disorders of sex development (DSD), gonadal dysgenesis, hypospadias, novel variants, NR5A1

## Abstract

**Rationale:**

Nuclear receptor subfamily 5 group A member 1 (NR5A1), also known as steroidogenic factor 1 (SF-1), is a master regulator of gonadal development and steroidogenesis. Pathogenic variants in NR5A1 are increasingly recognized as a significant cause of 46,XY disorders of sex development (DSD) and hypospadias, yet the mutational spectrum – particularly in pediatric populations – remains incompletely characterized, hindering early diagnosis and personalized management.

**Patient concerns:**

Four unrelated pediatric patients presented with a spectrum of genital anomalies: a 2-year-old 46,XY male with penoscrotal hypospadias; an 8-year-old 46,XY boy with micropenis and delayed puberty; a 5-month-old 46,XX infant with virilized genitalia and inguinal gonads; and a 2-year-old 46,XY child with severe genital ambiguity. Families expressed concerns about atypical genital appearance, uncertain sex assignment, future fertility, and the need for surgical or hormonal intervention.

**Diagnoses:**

All patients were diagnosed with DSD based on clinical, hormonal, and histopathological findings. Whole-exome sequencing identified four novel NR5A1 variants: a splice-site variant (c.1138 + 5G>A), a DNA-binding domain missense variant (c.308G>A; p.Arg103Gln), a deep intronic variant (c.990 + 20C>T), and a ligand-binding domain missense variant (c.1352T>G; p.Leu451Arg). Biochemical profiles consistently showed hypergonadotropic hypogonadism (elevated follicle-stimulating hormone, low testosterone/dehydroepiandrosterone sulfate), and gonadal biopsies confirmed dysgenesis.

**Interventions:**

One patient (Case 1) underwent a novel one-stage sealed Y-shaped penile foreskin vascular protection surgery for hypospadias repair. The other patients received multidisciplinary care, including genetic counseling, endocrine monitoring, and planning for future hormone replacement or reconstructive surgery as needed.

**Outcomes:**

The surgical intervention in Case 1 achieved excellent cosmetic and functional outcomes with preserved vascular integrity. All patients remain under long-term follow-up. No complications related to interventions were reported during the observation period.

**Lessons:**

This case series expands the pathogenic landscape of NR5A1 and demonstrates its capacity to disrupt sexual development across diverse karyotypes (46,XY and 46,XX). Early genetic diagnosis enables accurate classification, informs surgical and endocrine decision-making, guides gonadoblastoma surveillance, and facilitates family counseling. NR5A1 should be included in first-tier genetic testing for children with unexplained hypospadias or DSD, especially when accompanied by biochemical evidence of primary gonadal failure.

## 1. Introduction

Nuclear receptor subfamily 5 group A member 1 (also known as steroidogenic factor 1, SF-1) (NR5A1) is a key transcription factor that plays an indispensable role in gonadal development, steroidogenesis, and the differentiation of the reproductive system.^[1,2]^ As a central regulator of these processes, NR5A1 governs the expression of numerous genes involved in sex determination, steroid hormone biosynthesis, and maintenance of gonadal function. Pathogenic variants in NR5A1 have been increasingly recognized as significant contributors to a spectrum of disorders, including disorders of sex development (DSD), hypospadias, and gonadal dysfunction.^[3,4]^ These conditions often manifest with phenotypic heterogeneity, ranging from mild genital anomalies to severe gonadal dysgenesis, underscoring the complex interplay between genetic factors and clinical outcomes.^[4,5]^

Despite advances in understanding the molecular basis of NR5A1-related disorders, the full mutational spectrum and functional consequences of novel variants remain incompletely characterized. Whole-exome sequencing (WES) has emerged as a powerful tool for identifying rare and novel genetic variants associated with Mendelian disorders, providing critical insights into genotype–phenotype correlations. In this study, we utilized WES to identify previously unreported NR5A1 variants in 4 unrelated patients presenting with varying degrees of hypospadias, ambiguous genitalia, and gonadal dysgenesis. Through comprehensive endocrine evaluations and histopathological analyses, we aimed to elucidate the functional impact of these variants on gonadal development and steroidogenesis.

Additionally, we highlight the clinical management of 4 patient who underwent a novel 1-stage sealed Y-shaped penis foreskin vascular protection surgery developed by our team,^[6]^ demonstrating its potential as an effective therapeutic approach for genital reconstruction in patients with NR5A1-related disorders. Our findings not only expand the known mutational landscape of NR5A1 but also emphasize its critical role in DSD and hypospadias. These results provide valuable insights for improving clinical diagnosis, facilitating genetic counseling, and guiding personalized therapeutic strategies for affected individuals.

## 2. Case presentations

### 2.1. Case 1: A 2-year-old 46,XY male with hypospadias and a novel splice-site variant (c.1138 + 5G > A)

**Figure 1.**
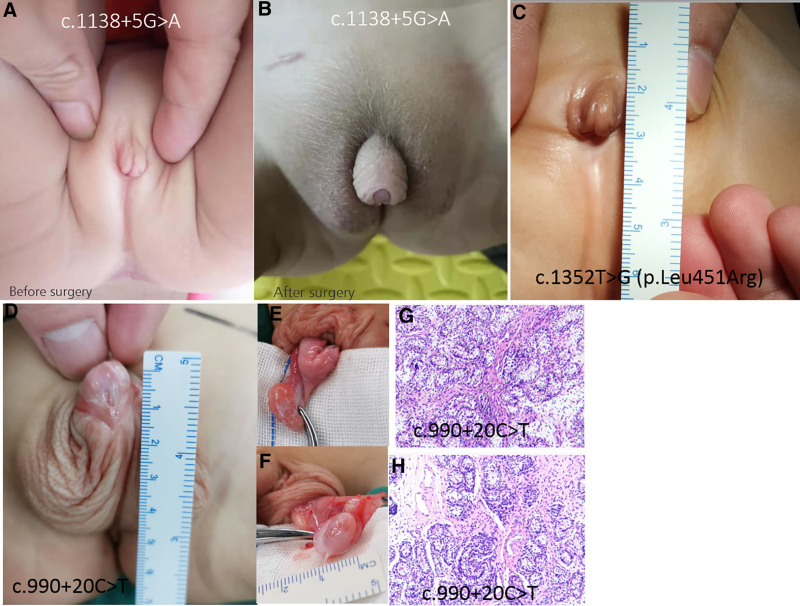
Preoperative and postoperative genital morphology in patients with NR5A1-related disorders. (A–B) Representative images of Case 1 (46,XY male, 2 years old). (A) Preoperative view showing hypospadias with a ventral urethral opening and penile curvature. (B) Postoperative outcomes following 1-stage sealed Y-shaped penile foreskin vascular protection surgery. This novel surgical technique achieved anatomical correction, improved penile alignment, and preserved vascular integrity. (C) Representative images of Case 4 (c.1352T > G [p.Leu451Arg]) demonstrating ambiguous genitalia with a short penis. (D–H) Representative images of Case 4: (D–E) external appearance of the gonads; (G–H) histological analysis of the gonads using hematoxylin and eosin (HE) staining.

A 2-year-old phenotypic male presented with hypospadias, characterized by a ventrally located urethral meatus, mild penile curvature, and abnormal foreskin hooding. Endocrine evaluation revealed biochemical evidence of primary gonadal failure, with low serum testosterone and elevated follicle-stimulating hormone (FSH) levels. WES identified a novel heterozygous variant in *NR5A1*: c.1138 + 5G > A, located 5 nucleotides upstream of the exon 6–intron 6 boundary. This variant was absent from population databases (gnomAD, 1000 Genomes, ESP, dbSNP) and predicted by SpliceAI (Δscore = 0.93) to severely disrupt canonical mRNA splicing, likely leading to exon skipping or a truncated, nonfunctional SF-1 protein. Testicular biopsy confirmed gonadal dysgenesis, showing markedly underdeveloped seminiferous tubules with absent spermatogenesis. The patient underwent our team’s novel 1-stage sealed Y-shaped penile foreskin vascular protection surgery, which successfully corrected the urethral defect and penile curvature while preserving vascular supply. Postoperative recovery was uneventful, with excellent cosmetic and functional outcomes observed at follow-up (Fig. 1A and B). Long-term endocrine and urologic monitoring is ongoing.

### 2.2. Case 2: An 8-year-old 46,XY male with delayed puberty and a DNA-binding domain mutation (c.308G > A; p.Arg103Gln)

An 8-year-old boy presented with ambiguous genitalia, including a micropenis and bilateral hypoplastic testes, along with clinical signs of delayed puberty. Hormonal profiling showed elevated FSH and low dehydroepiandrosterone sulfate (DHEA-S), consistent with hypergonadotropic hypogonadism. Genetic analysis revealed a novel missense variant, c.308G > A, resulting in the amino acid substitution p.Arg103Gln within the highly conserved DNA-binding domain of SF-1. This domain is critical for sequence-specific DNA recognition; thus, the substitution is predicted to impair transcriptional activation of downstream target genes such as *SOX9* and steroidogenic enzymes. Histopathological examination of the testes demonstrated poorly developed seminiferous tubular epithelium, confirming gonadal dysgenesis. No surgical intervention was performed at the time of reporting; the patient is under multidisciplinary management including endocrine follow-up and future consideration of testosterone replacement therapy at pubertal age.

### 2.3. Case 3: A 5-month-old 46,XX infant with atypical male genitalia and a deep intronic variant (c.990 + 20C > T)

This infant, karyotyped as 46,XX, was referred for evaluation of atypical male external genitalia at birth, including penoscrotal hypospadias, penile curvature, and a right-sided inguinal hernia. Physical examination revealed bilateral gonadal masses in the inguinal canals. Remarkably, whole-exome sequencing did not identify pathogenic variants in other known DSD genes but detected a novel deep intronic variant in *NR5A1*: c.990 + 20C > T. In silico analysis (SpliceAI Δscore = 0.01) indicated minimal impact on canonical splicing, and the variant is not predicted to directly alter protein coding. However, given the striking phenotype (including histopathological confirmation of “partial testicular-like structures” in the right gonad and hypoplasia of the left gonad) and the absence of alternative genetic explanations, we cannot exclude a potential pathogenic role for this variant. It may exert its effect through non-canonical mechanisms, such as altering splicing regulatory elements or chromatin architecture, leading to aberrant activation of testis-determining pathways in a 46,XX context. This case highlights the capacity of *NR5A1* variants to override chromosomal sex and underscores the importance of considering non-coding variants in genetically unresolved DSD cases (Fig. 1D–H).

### 2.4. Case 4: A 2-year-old 46,XY male with severe genital ambiguity and a ligand-binding domain (LBD) mutation (c.1352T > G; p.Leu451Arg)

A 2-year-and-1-month-old child with 46,XY karyotype presented with severely ambiguous genitalia: a clitoris-like phallus measuring approximately 1 cm, bifid scrotum, and bilateral inguinal gonads of asymmetric size. Endocrine evaluation showed markedly elevated FSH (15.61 mIU/mL) and low DHEA-S (8.8 μg/dL), indicating impaired gonadal steroidogenesis. Genetic testing identified a novel missense variant, c.1352T > G, resulting in p.Leu451Arg within the LBD of SF-1. The LBD is essential for protein–protein interactions and transcriptional co-activator recruitment; thus, this substitution likely compromises SF-1’s transactivation potential without necessarily affecting DNA binding. Gonadal histology from both sides revealed underdeveloped seminiferous tubules with interstitial edema, consistent with early-stage dysgenesis. The family is currently undergoing genetic counseling, and a multidisciplinary team is developing a long-term care plan that may include future reconstructive surgery and hormone replacement therapy (Fig. 1C).

Collectively, this case series demonstrates that heterozygous pathogenic or likely pathogenic variants in NR5A1 (spanning splice-site, DNA-binding, ligand-binding, and deep intronic regions) can disrupt gonadal development and steroidogenesis across diverse karyotypic and phenotypic spectra. All 4 patients exhibited biochemical hallmarks of primary gonadal failure (elevated FSH, low testosterone/DHEA-S) and histopathological evidence of gonadal dysgenesis, ranging from absent spermatogenesis (Case 1) to underdeveloped seminiferous tubules with interstitial edema (Case 4). Notably, the phenotypic manifestations extended from isolated hypospadias in a 46,XY male (Case 1) to severe genital ambiguity (Case 4) and, remarkably, to virilized genitalia in a 46,XX infant (Case 3), illustrating NR5A1’s capacity to override chromosomal sex. Furthermore, our successful application of a novel 1-stage Y-flap urethroplasty with vascular preservation in Case 1 highlights the feasibility of tailored surgical reconstruction in NR5A1-related DSD. These outcomes underscore the necessity of early genetic diagnosis to guide multidisciplinary management (including endocrine replacement, surgical planning, gonadoblastoma surveillance, and psychosocial support) thereby optimizing long-term health and quality of life for affected individuals.

## 3. Materials and methods

### 3.1. WES and bioinformatics analysis

Genomic DNA was extracted from 1.5 mL of peripheral blood collected from each participant. WES was performed using the Illumina NovaSeq 6000 platform (Saifu Biotechnology Co., Ltd, Suzhou, China). Exonic regions were captured using the IDT xGen Exome Research Panel, followed by library preparation and paired-end sequencing (2 × 150 bp). Raw sequencing data (>10 Gb per sample) with a Q30 base quality score ≥ 80% were retained for downstream analysis.

Raw sequencing reads (.bcl files) were converted to FASTQ format using bcl2fastq (Illumina). High-quality reads were aligned to the human reference genome (GRCh38/hg38) using BWA-MEM. SAM files were processed with SAMtools and Picard Tools for sorting, duplicate marking, and BAM file generation. Local realignment around indels and variant calling were performed using the Genome Analysis Toolkit (GATK v4.x) following best practices.

Resulting variant call format (.vcf) files were annotated using ANNOVAR to determine functional consequences, population frequencies, and clinical relevance. Variant filtering was conducted based on the following criteria:

1. Retention of exonic and splice-site variants, with prioritization of non-synonymous, frameshift, or canonical splice-site alterations.
2. Exclusion of variants with minor allele frequency ≥ 0.05 in population databases, including gnomAD, ExAC (EAS and ALL populations), and 1000 Genomes Project.
3. Cross-referencing with clinical databases such as ClinVar, HGMD, OMIM, and dbSNP to assess known pathogenicity.
4. In silico prediction of functional impact using multiple algorithms: SIFT, PolyPhen-2, LRT, MutationTaster, and FATHMM.

All candidate pathogenic variants were interpreted according to the American College of Medical Genetics and Genomics guidelines and correlated with the patient’s clinical phenotype. To confirm the accuracy of WES findings, all reported variants were validated by Sanger sequencing. When available, parental samples were also analyzed to determine the inheritance pattern (de novo or inherited).

### 3.2. Ethical review

The study was approved by the Ethics Committee of Changsha Maternal and Child Health Hospital (Approval No.: Quick EC-20250430-10). Written informed consent was obtained from the legal guardians of all 4 minor patients for genetic testing, publication of anonymized clinical data, and photographic documentation.

## 4. Discussion

The identification of 4 novel *NR5A1* variants in our cohort of patients with DSD and hypospadias provides critical insights into the molecular etiology and phenotypic spectrum of this clinically heterogeneous condition. Rather than representing a simple Mendelian disorder, *NR5A1*-related DSD exemplifies the complexity of human sexual development, where a single transcription factor sits at the nexus of multiple developmental pathways, and its disruption can lead to a wide array of phenotypic outcomes (from 46,XY complete gonadal dysgenesis to 46,XX testicular DSD).^[7]^ Our findings underscore that the primary value of identifying such variants lies not in enabling “personalized therapy” in a narrow pharmacological sense, but in elucidating the underlying pathogenesis, refining diagnostic classification, and guiding evidence-based, multidisciplinary clinical management.

The pathogenic mechanisms of these variants converge on 2 fundamental biological processes: gonadal differentiation and steroid hormone biosynthesis. The splice-site variant c.1138 + 5G > A (Case 1) and the DNA-binding domain missense variant p.Arg103Gln (Case 2) are predicted to cause loss-of-function, leading to impaired activation of key downstream targets such as *SOX9* and *AMH*. This results in testicular dysgenesis, as confirmed histologically by underdeveloped seminiferous tubules, and consequent androgen deficiency that manifests as hypospadias and micropenis. In contrast, the 46,XX patient (Case 3) carrying the deep intronic variant c.990 + 20C > T presents a fascinating counterpoint. Despite minimal predicted impact on canonical splicing, the presence of testicular-like tissue and virilized genitalia strongly suggests a potential gain-of-function or neomorphic effect (perhaps through the creation of a cryptic regulatory element or alteration of chromatin architecture) that aberrantly activates the male developmental pathway in the absence of a Y chromosome. This case vividly illustrates how *NR5A1* dysfunction can override chromosomal sex, contributing to the remarkable phenotypic heterogeneity observed in DSD.

The LBD variant p.Leu451Arg (Case 4) further highlights the domain-specific consequences of *NR5A1* mutations. While likely preserving DNA-binding capacity, this substitution is predicted to disrupt protein–protein interactions or cofactor recruitment, leading to defective transactivation of steroidogenic enzymes such as *CYP17A1* and *HSD3B2*. This is biochemically reflected in the consistent endocrine profile across our cohort: low androgen precursors (testosterone, DHEA-S) coupled with elevated gonadotropins (FSH), indicative of primary gonadal failure. The resulting androgen insufficiency during critical fetal developmental windows directly explains the spectrum of external genitalia malformations observed in all 4 patients.

Clinically, the significance of these findings is threefold. First, they expand the mutational landscape of *NR5A1*, reinforcing its status as a major genetic contributor to both 46,XY and 46,XX DSD. This argues strongly for the inclusion of *NR5A1* in 1st-tier genetic testing panels for any child presenting with unexplained hypospadias, ambiguous genitalia, or gonadal dysgenesis, especially when accompanied by biochemical evidence of hypergonadotropic hypogonadism. Second, a molecular diagnosis provides crucial prognostic information. For example, the identification of gonadal dysgenesis mandates long-term surveillance for gonadoblastoma and informs decisions regarding prophylactic gonadectomy. Third, while “personalized therapy” in the sense of gene-targeted drugs is not currently feasible, genetic diagnosis enables truly personalized clinical management (guiding decisions on hormone replacement timing, surgical reconstruction strategies) (as exemplified by our novel Y-flap technique in Case 1), and psychosocial support tailored to the specific etiology and expected trajectory of the condition.

In conclusion, this case series deepens our understanding of how distinct *NR5A1* lesions (whether affecting splicing, DNA-binding, or protein interaction domains) disrupt the delicate orchestration of human sex development. The clinical heterogeneity observed is not a confounding factor but a direct reflection of the gene’s central role in this process. Future functional studies, such as in vitro splicing assays or transcriptional reporter assays, are urgently needed to definitively validate the pathogenicity of these novel variants, particularly the deep intronic change in Case 3. Ultimately, integrating genetic diagnosis into the clinical workflow for DSD is not about tailoring drugs, but about providing families with a clear etiological explanation, enabling anticipatory guidance, and facilitating a coordinated, mechanism-informed approach to lifelong care.

### 4.1. Limitations

This study has several limitations. First, as a case series, it lacks a control group and statistical power for genotype–phenotype correlation. Second, functional validation (e.g., splicing assays for c.1138 + 5G > A and c.990 + 20C > T, or transcriptional activity assays for c.1352T > G) was not performed due to technical constraints. Third, long-term follow-up data on fertility and cancer risk are not yet available. Future multicenter studies with functional analyses are needed to confirm the pathogenicity of these novel variants and refine clinical management guidelines.

## Acknowledgments

We sincerely thank the patients and their families for their trust, cooperation, and invaluable participation in this study. We also gratefully acknowledge Ni Gan for their expert clinical assistance and valuable contributions to patient management and data collection. We extend our appreciation to all members of the clinical, surgical, and laboratory teams whose dedicated efforts were essential to the successful completion of this research.

## Author contributions

**Conceptualization:** Jiancheng Zu, Qingqing Hu.

**Data curation:** Jiancheng Zu, Sifeng Wang, Qingqing Hu.

**Formal analysis:** Xiangwen Peng, Qingqing Hu.

**Funding acquisition:** Xiangwen Peng, Qingqing Hu.

**Investigation:** Xiangwen Peng.

**Software:** Xiangwen Peng.
